# Interplay of cell death signaling pathways mediated by alternating magnetic field gradient

**DOI:** 10.1038/s41420-018-0052-7

**Published:** 2018-04-27

**Authors:** De Wei Wong, Wei Liang Gan, Yuan Kai Teo, Wen Siang Lew

**Affiliations:** 1 School of Physical and Mathematical Sciences, Nanyang Technological University, 21 Nanyang Link, Singapore, 637371 Singapore; 2 School of Biological Sciences, Nanyang Technological University, 60 Nanyang Drive, Singapore, 637551 Singapore

## Abstract

The ability to control or manipulate the pathways leading to cell death plays a pivotal role in cancer treatment. We demonstrate magneto-actuation of magnetic nanoparticles (MNPs) to induce different cell death signaling pathways, exemplifying the intricate interplay between apoptosis and necrosis. In vitro cell experiments show the cell viabilities decreases with increasing field strength and is lower in cells treated with low aspect ratio MNPs. In a strong vertical magnetic field gradient, the MNPs were able to apply sufficient force on the cell to trigger the intracellular pathway for cell apoptosis, thus significantly reducing the cell viability. The quantification of apoptotic and necrotic cell populations by fluorescence dual staining attributed the cell death mechanism to be predominantly apoptosis in a magnetic field gradient. In contrast, the MNPs in an alternating magnetic field gradient can effectively rupture the cell membrane leading to higher lactate dehydrogenase leakage and lower cell viability, proving to be an effective induction of cell death via necrosis.

## Introduction

In recent years, magnetic nanoparticles (MNPs) have rapidly gained traction in the biomedical fields as magnetic resonance imaging contrast agents^1,2^, biosensors^3,4^ and controlled drug delivery^5,6^. In addition, MNPs with engineered magnetic properties and high biocompatibility have been shown to be a promising candidate for cancer treatment. A well-established method for treating cancerous tumors is magnetic hyperthermia, which uses localized heat generation by the interaction of the MNPs in a high-frequency alternating magnetic field, to trigger cancer cell apoptosis and tumor regression^7–10^. The magnetic hysteresis of the MNPs result in energy dissipated as thermal energy that induces a rise in temperature to a range of 41‒43 °C. However, the exposure of tumor tissue to temperatures above 43 °C causes necrosis of cancer cells^11,12^. In the case of low-frequency alternating magnetic fields, the heat generated by the MNPs becomes negligible, but the mechanical stress exerted on the cells can cause mechanical disruption or compromise the integrity of the cell membrane, inducing necrosis^13–15^. It has also been established in in vitro cell destruction experiments that spin-vortex-mediated stimulus by MNPs was sufficient for the initiation of programmed cell death^16–18^.

Multiple cell death pathways can be observed simultaneously in cell cultures or tissues exposed to different types of MNPs and magnetic field configurations. Apoptosis is a form of programmed cell death characterized by morphological features, such as reduction of cell volume, membrane blebbing and formation of apoptotic bodies^19,20^. It is vital for normal development, homeostasis and functioning of the immune system, and anti-inflammatory reactions. Necrosis is a form of unprogrammed cell death arising from external perturbations with the release of intracellular contents after cell membrane damage, causing inflammation.

In this work, we examined the force exerted by MNPs with different aspect ratios under both uniform and non-uniform magnetic fields. The magnetic field generates a magnetic torque on the MNPs, which in turns exerts a force onto the HeLa cells inducing apoptosis or necrosis. Acridine Orange and Ethidium Bromide (AO/EB) fluorescence dual staining, which quantifies the live, apoptotic and necrotic cell populations, attributes the cell death mechanism to be predominantly apoptosis by the uniform magnetic field or field gradient (FG). In an alternating magnetic field gradient (AFG), the MNPs oscillates with a force sufficient to mechanically rupture the cell membrane. The AO/EB dual staining reveals an increase in necrotic cell population coupled with higher lactate dehydrogenase (LDH) leakage and greater reduction in cell viability, indicating cell necrosis.

## Results and discussion

### Uniform magnetic field

**Fig. 1:**
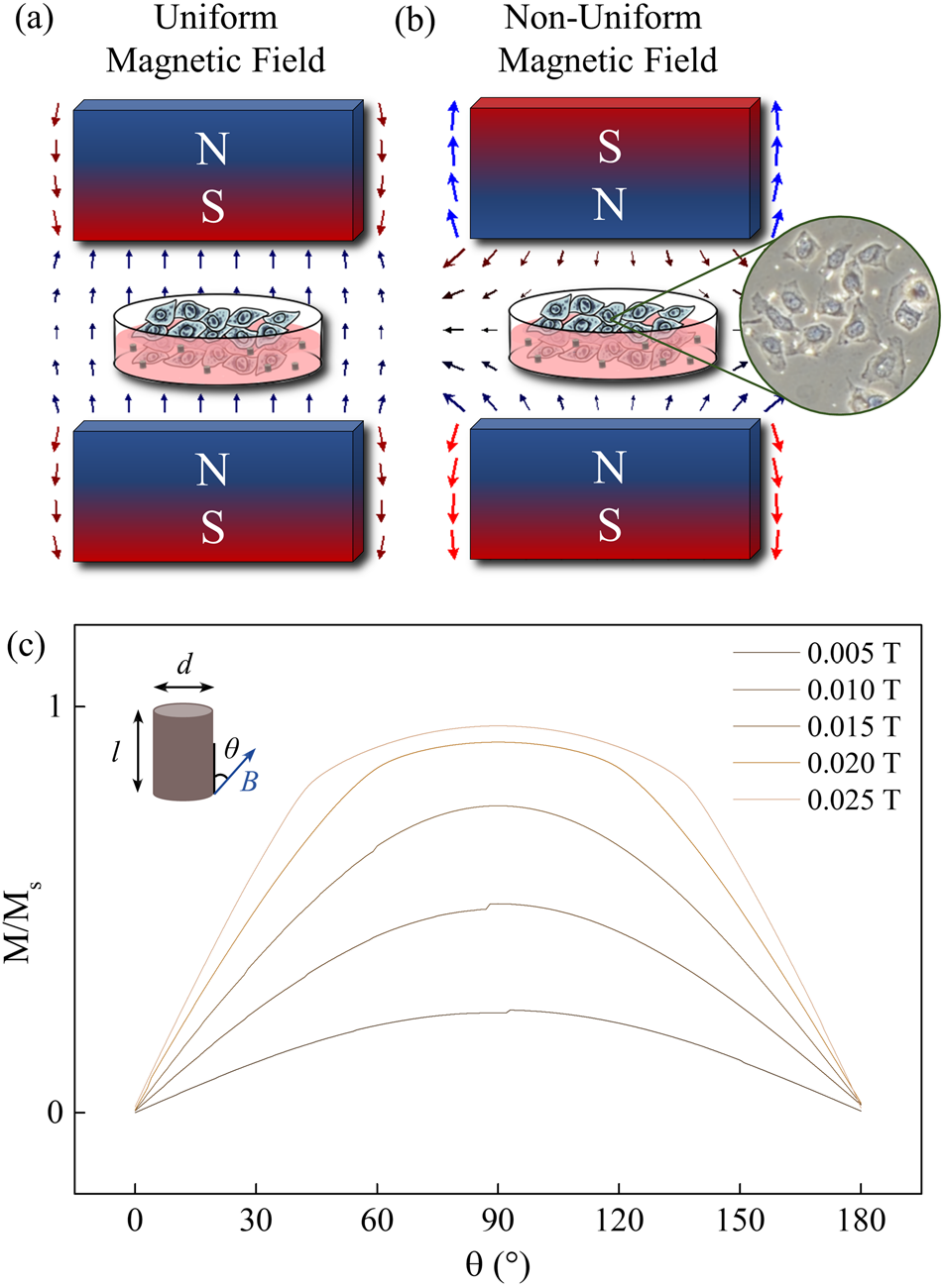
**a**, **b** Experimental setup of the electromagnetic coils employed to produce a vertically oriented magnetic field with two configurations; uniform magnetic field (i.e. zero gradient) and non-uniform magnetic field with a vertical magnetic field gradient. **c** MNP magnetization *M*/*M*_s_ in an applied magnetic field (*B* = 0.005‒0.025 T) with angular dependence (*θ* = 0‒180°)

A pair of electromagnetic coils was employed to create a vertically oriented magnetic field with two configurations; uniform magnetic field (i.e. zero gradient) and non-uniform magnetic field with a vertical magnetic FG (Fig. 1a, b). An infrared thermometer was used to remotely monitor the cell culture medium and kept at 23.0 ± 0.5 °C, eliminating any contributions from magnetic hyperthermia. In a uniform magnetic field, the MNP experiences a magnetic torque that rotates it so that its net magnetic moments are aligned to the field direction. The relationship between the magnetic torque and the applied magnetic field is given by $\left| {\mathbf{\tau }} \right| = \left| {{\mathbf{m}} \times {\mathbf{B}}} \right| = {\mathrm {MVBsin}}\theta$, where ***m*** is magnetic moment and *M* is the magnetization of the MNP in the applied magnetic field ***B***, the volume *V* of the MNP is given as$\pi \left( {\frac{d}{2}} \right)^2l$, and *θ* is the angle between the long axis of the MNP and ***B***. The magnitude of the force acting on the HeLa cells can be obtained by calculating the magnetic torque exerted on the edge of the MNP, $F = \frac{{2\left| {\mathbf{\tau }} \right|}}{l} = M\frac{{\pi d}}{2}^2B{\mathrm {sin}}\theta$. The angular dependence of ***M*** is obtained by applying a rotating constant magnitude uniform magnetic field ***B*** with respect to MNP long axis from *θ* = 0 to 180° (Fig. 1c). A weak magnetic field of *B* = 0.005‒0.025 T was applied, which is inadequate to influence the magnetization configurations of the MNPs, but sufficient for inducing the spatial rotation of the MNPs. The MNPs are first magnetically saturated by applying a strong magnetic field of 0.1 T. Upon relaxation, an anticlockwise vortex and a clockwise vortex form at the ends of the MNP, and are connected by a third vortex nucleated at the center of the MNP^17,21^. As the weak magnetic field rotates from 0° to 180°, ***M*** is observed to increase and saturate when the field is perpendicular (*θ* = 90°), from which the maximum torque can be obtained.

**Fig. 2:**
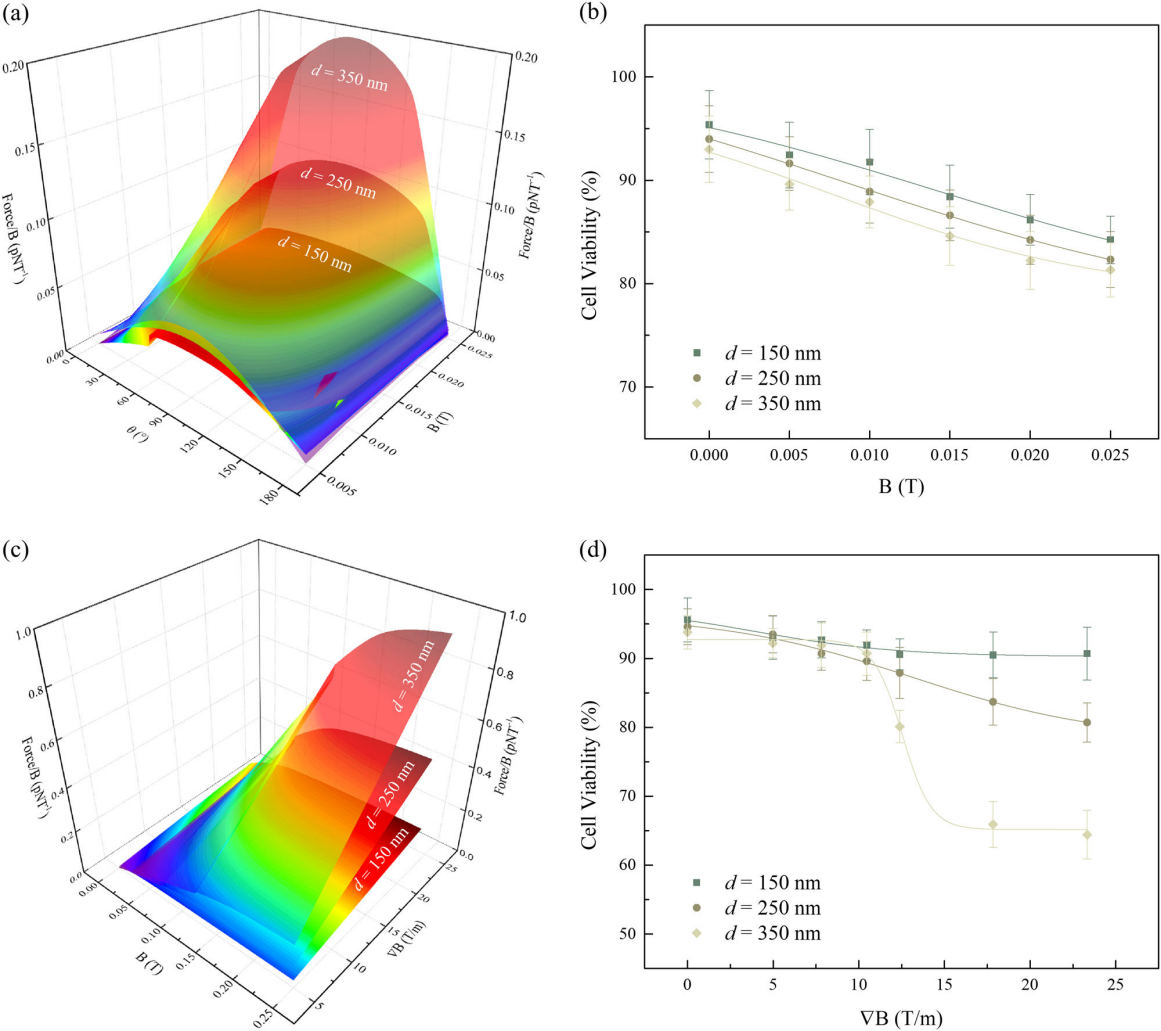
**a** Magnitude of force exerted by MNPs (*d* = 150, 200 and 350 nm), calculated by the torque exerted on the edge of the MNP, with respect to the angle *θ* and magnetic field strength ***B***. **b** Cell viability of HeLa cells after uniform magnetic field treatment of *B* = 0.005‒0.025 T. **c** Magnitude of force acting on MNPs (*d* = 150, 200 and 350 nm), proportional to the magnetic field gradient. **d** Cell viability of HeLa cells after non-uniform magnetic field treatment of ∇*B* *=* 0‒23.3 T/m

The magnitude of the force (*F*) exerted with respect to applied field strength (*B*) for various field angles (*θ*) shows the maximum force for MNPs with diameters *d* = 150, 250 and 350 nm to be 0.041 pN, 0.11 pN and 0.20 pN, respectively (Fig. 2a). While the physical rupture of the cell membrane requires a force of ~100 pN^22–24^, a force of 0.5 pN is sufficient for the activation of mechanosensitive ion channels mediated by mechanical stimuli, leading to apoptosis^25,26^. To observe the cellular response to the MNPs in an uniform magnetic field, the HeLa cells were exposed to a similar amplitude field of *B* = 0.005‒0.025 T, with a concentration of 0.1 mg/ml of MNPs, for a period of 10 min each. The cell viabilities were observed to decrease gradually with increasing field strength, and is slightly lower in cells with low aspect ratio MNPs (Fig. 2b). The results from this assay demonstrate that the magnetic field strength and MNP aspect ratios has no immediate adverse effect, with cell viability greater than 80%. In the absence of magnetic field (*B* = 0 T), the cytotoxicity of the MNPs had minimal effect on cell viability, which is consistent with the known cytotoxicity rates of NiFe MNPs used in hyperthermia research with HeLa cells^27^.

### Non-Uniform Magnetic Field

By reversing the polarity of one electromagnetic coil, a non-uniform magnetic field with a FG is obtained. In a non-uniform magnetic field, a translational force acts on the MNPs, which is proportional to ∇*B*. The force acting on the MNPs is given by$F = ({\mathbf{m}} \cdot \nabla ){\mathbf{B}} = M\frac{{\pi d^2l}}{4}\nabla B$. The maximum force for *d* = 150, 250 and 350 nm MNPs were calculated to be 0.18 pN, 0.49 pN and 0.97 pN, respectively (Fig. 2c). The cell viability also exhibits similar trends after the FG treatment (∇*B* *=* 0‒23.3 T/m). A significant reduction in cell viability was observed after the HeLa cells were treated with low aspect ratio MNPs at larger gradients, ∇*B* > 12.4 T/m (Fig. 2d). The micromagnetic simulations shows that only the low aspect ratios MNPs were able to deliver a force surpassing the limit required to trigger cell apoptosis, which is also substantiated in a 20% increase in efficacy in reduction of cell viability as compared to high aspect ratios MNPs. The force exerted by the magnetic torque is limited by an upper boundary due to magnetic saturation, while a greater translational magnetic force can be generated by larger directional derivatives in the applied magnetic field.

### Alternating Magnetic FG

In the FG configuration, magnetic field is applied in a single direction using DC pulses. While in the alternating magnetic field gradient (AFG), the polarity of the electromagnetic coils was constantly reversed by AC pulses, which inverses the direction of the magnetic FG periodically. Thus, the MNPs undergo a lateral back and forth oscillation with a dependency on the frequency and magnitude of the AC pulses. The alternating force induces movement of the MNPs in two opposite directions, downwards into the cells and upwards away from the cells. To examine the effectiveness of the treatments, the HeLa cells were exposed to both FG and AFG configurations for a range of frequencies between 0.17‒3.33 Hz over a period of 10 min each.

**Fig. 3:**
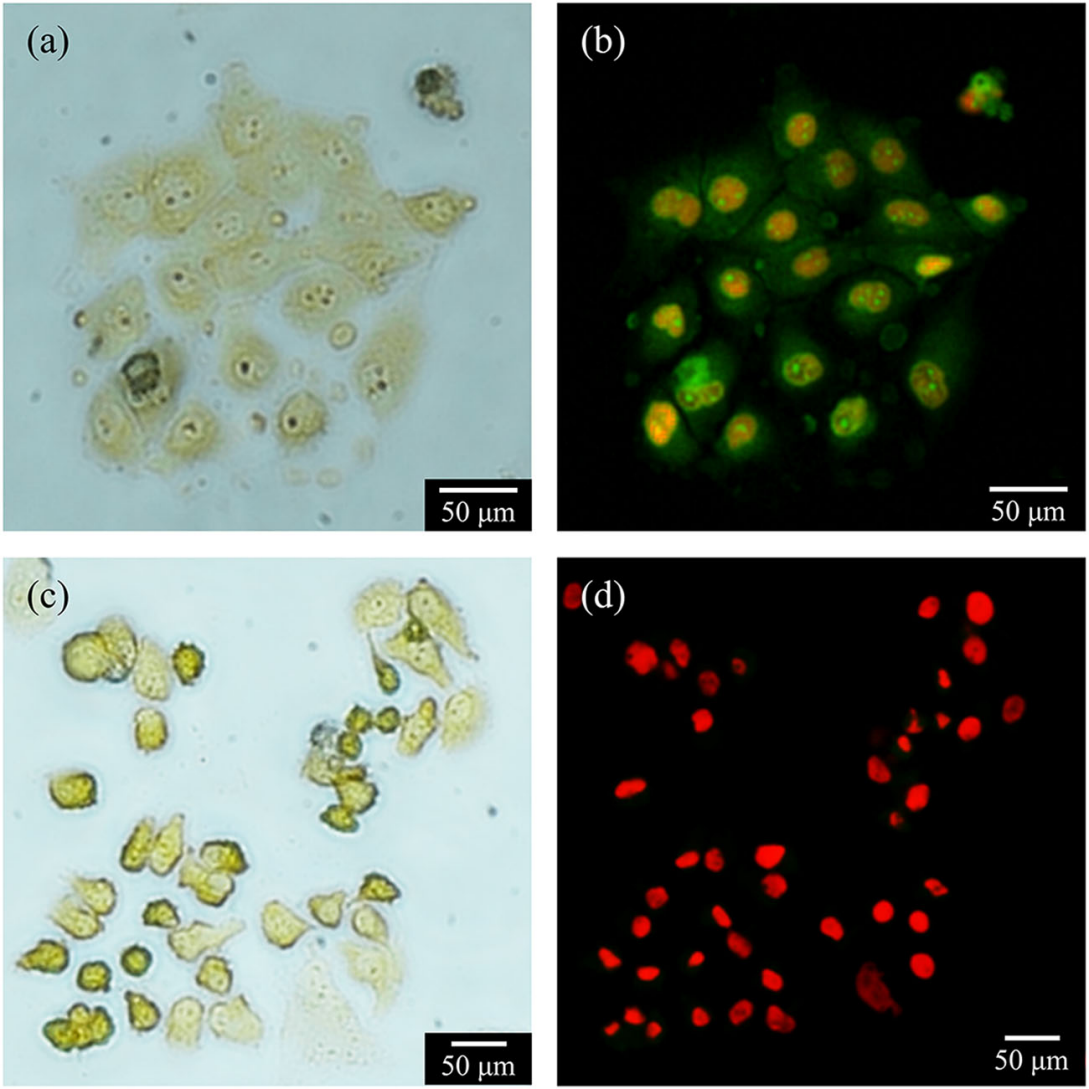
Fluorescence microscopy images of HeLa with AO/EB dual staining. **a**, **b** Treatment with magnetic field gradient (FG) shows membrane blebbing, fragmented chromatin and formation of apoptotic body, indicating apoptosis. **c**, **d** Treatment with alternating magnetic field gradient (AFG) shows an unapparent outline of the cells, rupture of both nuclear and plasma membranes, indicating necrosis

**Fig. 4:**
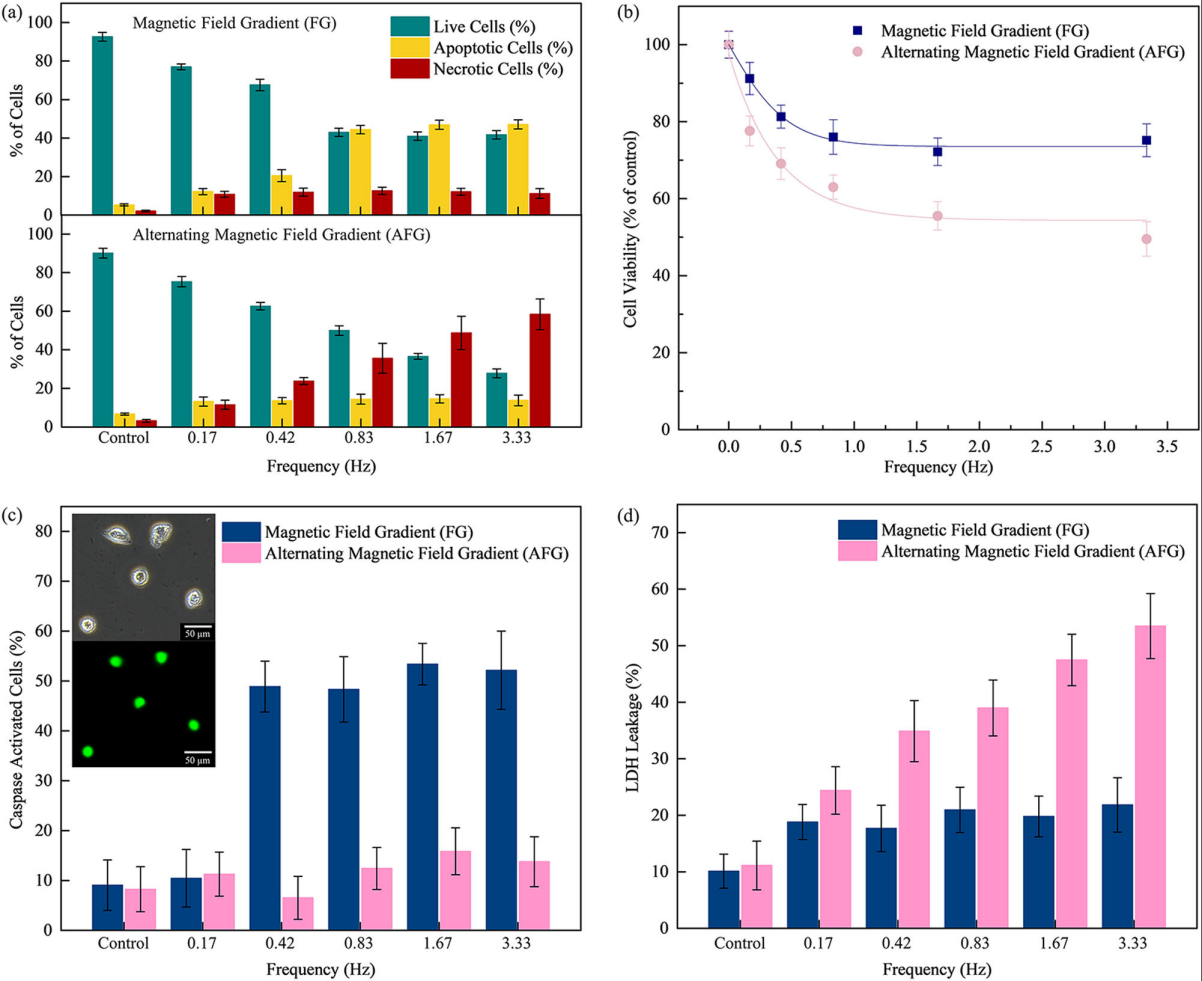
**a** The quantification cell populations in the control, magnetic field gradient (FG) and alternating magnetic field gradient (AFG) groups. The results are displayed as the mean percentage (%) of live, apoptotic and necrotic cell populations (±SD, *p* < 0.05). **b** Cell viability of HeLa cells after FG and AFG treatment for a range of frequencies between 0.17‒3.33 Hz. **c** Percentage of caspase-3/7-activated cells with displayed images of cells showing active caspase in bright green fluorescence. **d** Lactate dehydrogenase (LDH) leakage from the loss of cell membrane integrity in HeLa cells

Fluorescence microscopy images were obtained from AO/EB dual staining, which enabled the detection of changes in cell morphology by the differential uptake of the fluorescent DNA binding dyes. HeLa cells exposed to FG show orange apoptotic cells with fragmented chromatin, cell blebbing and the formation of apoptotic bodies, which implies that the FG configuration predominantly induces apoptosis (Fig. 3a, b). In contrast, the majority of HeLa cells exposed to AFG have ruptured nuclear and plasma membranes, exhibiting less defined cellular outlines (Fig. 3c, d). In addition, a uniformly red nucleus with organized structure was observed, indicating necrosis. The quantified data from AO/EB dual staining are presented in Fig. 4a. In the case of FG, the percentage of apoptotic cells population saturates with increasing frequencies. The highest percentage of apoptotic cells occurred at 3.33 Hz with 47% of the HeLa cell population. Switching to AFG, the percentage of necrotic cells increased near linearly as the frequency increases, while the percentage of apoptotic cells remains low at <15%. The highest percentage of necrotic cells (58%) was observed after the AFG treatment at 3.33 Hz, with the largest number of oscillation cycles. The in vitro study demonstrates that the cell membrane can be effectively disrupted by the lateral oscillations of the MNPs. In comparison, AFG was shown to be more effective at inducing HeLa cell death via largely necrosis.

To further investigate the cell death mechanisms induced by both magnetic FG configurations, the cell viability of both treatment methods were determined using the PrestoBlue assay. The results showed that FG and AFG led to a maximum reduction of 25 and 51% in cell viability, respectively (Fig. 4b). For FG, the cell viability remains relatively constant even when frequency is increased, reflecting a similar trend as the percentage of live cells from AO/EB dual staining. In contrast, AFG showed a more significant decrease in cell viability at all frequencies.

Caspase-3/7 are downstream executioner caspases associated with the apoptotic pathway that are responsible for apoptotic chromatin condensation and DNA fragmentation^28^. The activation of caspase-3/7 is thus a hallmark characteristic of apoptosis. The activity of caspase-3/7 in the HeLa cells were measured by CellEvent™ Caspase-3/7 Green Detection Reagent. The percentage of caspase-3/7-activated cells was substantially increased in FG from 9 to 53% at 3.33 Hz, a clear indication of induced apoptosis (Fig. 4c). The treatment with AFG showed minimal caspase-3/7 activity at all frequencies (<15%). Therefore, the activation of caspases-3/7 provided further evidence for the induction of apoptosis in response to the treatment with FG.

%When the cell membrane integrity is compromised or damaged, LDH, a cytosolic enzyme present in most cells, is released into the surrounding cell culture medium^29–31^. The inherent linearity of the Pierce LDH cytotoxicity assay kit allows it to accurately enumerate the number of necrotic cells in the cell culture medium^29^. The treatment with AFG reveals a continuous rise in LDH levels for increasing frequencies, while the treatment with FG showed insignificant changes in LDH levels at all frequencies (Fig. 4d). At 3.33 Hz, the LDH release was 381% higher than the untreated control cells. This is in accordance with the results from the AO/EB dual staining, which shows that the percentage of necrotic cells increases only for the treatment with AFG. A 24-h exposure to MNPs, without any magnetic field treatments, showed no significant increase in extracellular LDH in the cell culture medium. This ensures that the observed extracellular LDH is not due to stimulated LDH secretion by exposure to the MNPs, but due to the loss of cell membrane integrity^32^.

**Fig. 5:**
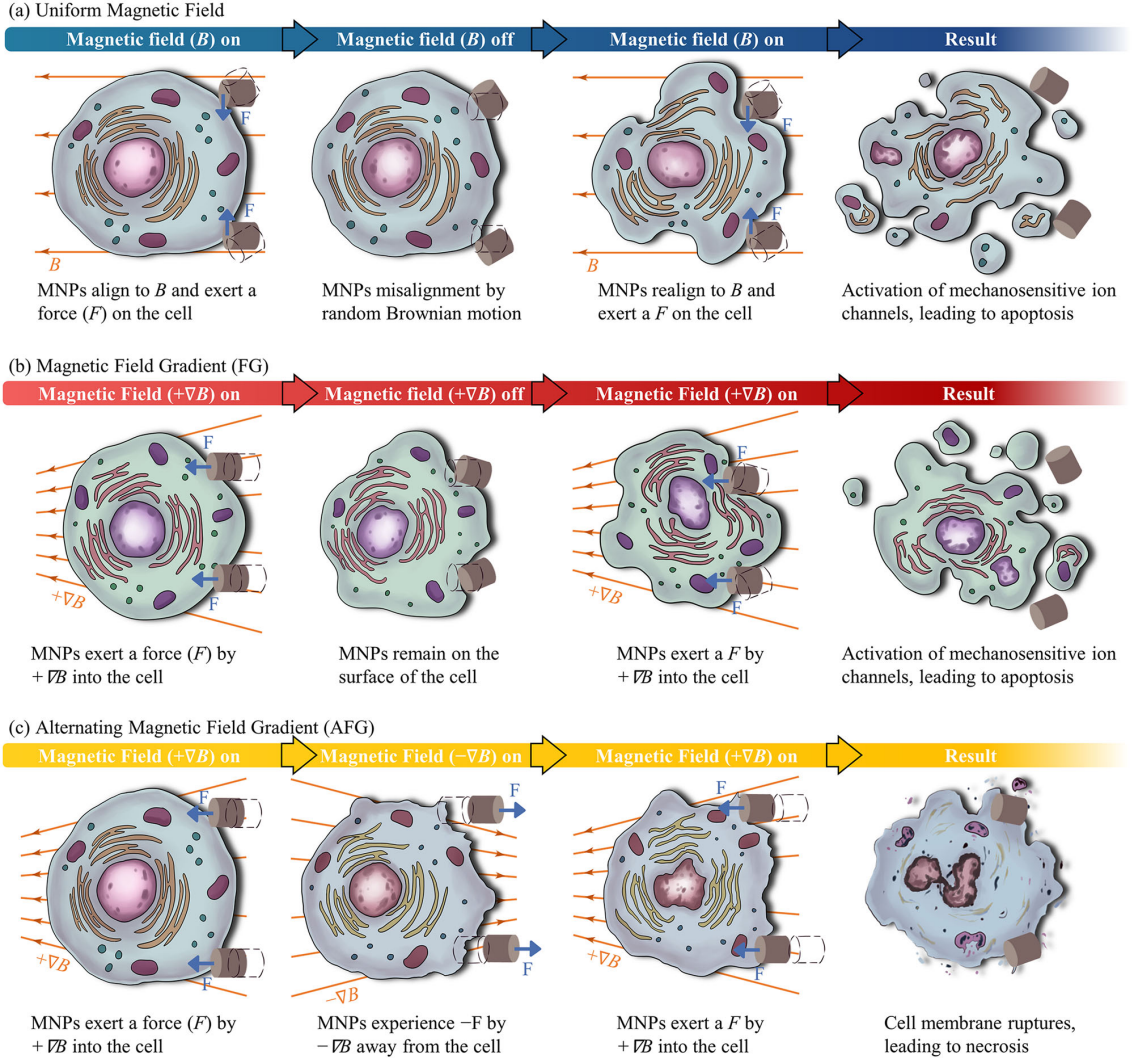
Schemes of the proposed cell death mechanisms for uniform and non-uniform magnetic fields. **a** Uniform magnetic field configuration utilizes the spatial rotation of MNPs to exert a force onto the cell, activating mechanosensitive ion channels, leading to cell apoptosis. **b** In magnetic field gradient (FG), MNPs experience a force by the one-dimensional linear field gradient downwards into the cells, only sufficient for mechanical stimuli-mediated cell apoptosis by the activation of mechanosensitive ion channels. **c** In alternating magnetic field gradient (AFG), the lateral oscillation of MNPs can mechanically rupture the cell membranes, triggering necrosis

The proposed cell death mechanisms for the uniform and non-uniform magnetic fields are summarized in Fig. 5. When the MNPs are exposed to a uniform magnetic field, it experiences a torque that aligns its net magnetic moments to the field direction by Brown relaxation (Fig. 5a). After the magnetic field is removed, random rotation Brownian motion causes random change in the orientation of MNPs. The uniform magnetic field configuration utilizes the spatial rotation of MNP, which exerts a force onto the cell, activating mechanosensitive ion channels, leading to cell apoptosis^33^. The activation of mechanosensitive ion channels as a result of cell membrane stretching, leads to the increase in intracellular calcium^34,35^. This prolonged exposure to high concentration of intracellular calcium triggers cell apoptosis^36–38^. In the cases of non-uniform magnetic fields, there is a translational force acting on the MNPs which is proportional to the magnetic FG. For FG, the MNPs experience a force by the one-dimensional linear magnetic FG into the cells. The force exerted by the MNPs on the cell is sufficient for mechanical stimuli-mediated cell apoptosis by the activation of mechanosensitive ion channels, but is insufficient to physically rupture the cell membrane (Fig. 5b). In contrast, AFG oscillates the MNPs laterally to induce mechanical damage to the cell membrane, leading to necrosis (Fig. 5c).

## Conclusion

In summary, we have demonstrated the ability to control the initiation of cell apoptosis or necrosis by magneto-actuation of MNPs. The force exerted by low aspect ratio MNPs on the cells is sufficient to induce cell apoptosis in a uniform magnetic field or FG. By introducing an AFG, the force exerted from oscillations of the MNPs is sufficient to physically rupture the cell membrane, leading to necrosis. The LDH activity in the cell culture medium begins to increase in parallel to the increase in necrotic cell populations measured by AO/EB dual staining. Hence, this remote magneto-actuation approach is a non-invasive and highly effective treatment method that can inhibit cancer cell proliferation by the induction of apoptosis or necrosis.

## Materials and methods

### Fabrication of NiFe MNPs

The MNPs were fabricated by using a combination of anodized aluminium oxide (AAO) template-assisted pulsed electrodeposition and differential chemical slicing techniques^17,21^. The Permalloy Ni_80_Fe_20_ MNPs were obtained with an electrolyte composition of 0.5 M nickel sulfate (NiSO_4_): 0.01 M iron sulfate (FeSO_4_). The length (*l*) of the MNPs is fixed by the high potential pulse durations at *l* = 500 nm. The diameter (*d*) of the MNPs is defined by the AAO template pore sizes, with *d* = 150‒350 nm.

### Cell Culture

HeLa cells were seeded into 96-well microtiter plate at 1 × 10^4^ cells/well and incubated in Dulbecco’s-modified Eagle’s medium supplemented with 4.5 g/L glucose, 2 mM l-glutamine, 10% fetal bovine serum and 1% penicillin/streptomycin maintained under a humidified atmosphere at 37 °C, 5% CO_2_.

### Cell Viability

The cell viability was assessed using the PrestoBlue assay kit, a fluorescent indicator of cell proliferation. The HeLa cells were incubated with PrestoBlue reagent at 37 °C, 5% CO_2_ for 2 h. The Tecan Infinite M200 PRO Microplate Reader was used to measure the absorbance values at 570 nm and 600 nm. Each experiment was performed in quadruplicate sets of experimental and control assays in a 96-well microtiter plate.

### Quantification of Cell Death

The cells were stained with AO/EB for the quantification of live, apoptotic and necrotic cell populations in the control and treated groups. AO is a cell-permeant nucleic acid binding dye that stains both viable and non-viable cells and emits green fluorescence. EB is a sensitive fluorescent dye that only stains cells with damaged membranes and emits red fluorescence. This fluorescence distinction between live, apoptotic and necrotic cells allows AO/EB dual staining to be a qualitative and quantitative evaluation of the cell proliferation and cell death effects in our treatments^39–42^. The cells were incubated with 20 μg/ml AO and 20 μg/ml EB fluorescent dyes at 37 °C, 5% CO_2_ for 30 min, and analyzed under a Nikon Eclipse Ti–S inverted microscope. A minimum of 300 cells were counted in each well to obtain the ratio between apoptotic and necrotic cells at each frequency between 0.17‒3.33 Hz and reported as a percentage of the total number of cell counted. Each experiment was performed in quadruplicate sets of experimental and control assays in a 96-well microtiter plate.

### Quantification of Caspase-3/7 Activation

CellEvent Caspase-3/7 Green Detection Reagent is a nucleic acid binding fluorescent dye. In apoptotic cells with activated caspase-3/7, the DEVD peptide is cleaved, which allows the dye to bind to DNA and emits bright green fluorescence^43–45^. The reagent was diluted into phosphate-buffered saline with 5% fetal bovine serum to a final concentration of 5 μM. The cell culture medium was removed and the cells were incubated with 100 μL of diluted reagent at 37 °C, 5% CO_2_ for 30 min. The Tecan Infinite M200 PRO Microplate Reader was used to measure the fluorescence signal at the absorption and emission values of 502 nm and 530 nm, respectively. Each experiment was performed in quadruplicate sets of experimental and control assays in a 96-well microtiter plate.

### Quantification of Cell Membrane Damage

The leakage of LDH into the cell culture medium from damaged cells is quantitatively measured by Pierce LDH cytotoxicity assay kit. After the magnetic field treatment, the cell culture supernatant is transferred to a new microplate and mixed with the reaction mixture reagent. After the microplate was incubated at room temperature for 30 min, the reactions were stopped by adding the stop solution. The LDH activity is determined by spectrophotometric absorbance at 490 nm. Each experiment was performed in quadruplicate sets of experimental and control assays in a 96-well microtiter plate. All reagents were purchased from Thermo Scientific.

### Statistical analysis

The results were represented as the mean ± standard deviation (SD). A *p* value of <0.05 was considered to be statistically significant.

### Micromagnetic Simulations Parameters

The magnetization dynamics of the MNPs in the various magnetic fields configurations were studied by means of a GPU-accelerated micromagnetic simulation program, MuMax3^46^. The material parameters for Permalloy Ni_80_Fe_20_ were used; saturation magnetization *M*_s_ = 860 × 10^3^ A/m, exchange stiffness constant *A*_ex_ = 1.3 × 10^−11^ J/m, zero magneto-crystalline anisotropy *k* = 0, and Gilbert damping constant *α* = 0.01^47–49^. A cell size of 5 nm × 5 nm × 5 nm was used for all simulations, which is sufficiently small as compared to the exchange length.
